# CSACI position statement: prescribing sublingual immunotherapy tablets for aeroallergens

**DOI:** 10.1186/s13223-017-0225-6

**Published:** 2018-01-09

**Authors:** J. Quirt, R. Gagnon, A. K. Ellis, H. L. Kim

**Affiliations:** 1 McMaster University, Hamilton, Canada; 2 Laval University, Quebec City, Canada; 3 Queens University, Kingston, Canada; 4 Western University, London, Canada; 5 525 Belmont Ave West, Suite 205, Kitchener, ON N2M 5E2 Canada

## Background

Allergic rhinitis is estimated to affect 20–25% of Canadians and has a significant impact on quality of life, with many patients reporting inadequate control of their symptoms [1]. Mainstays of treatment for allergic rhinitis include avoidance, intranasal steroids, oral antihistamines and leukotriene receptor antagonists [2]. Specific immunotherapy offers disease-modifying treatment for those uncontrolled by, intolerant to, or averse to pharmacotherapy [3].

Currently two types of aeroallergen immunotherapy are used in clinical practice: subcutaneous immunotherapy (SCIT) and sublingual immunotherapy (SLIT). SLIT was first accepted as an alternative to SCIT by the WHO in 1998, and was then introduced into the ARIA guidelines [4, 5]. While SLIT has been available in Europe for some time, Canada first approved a sublingual grass immunotherapy tablet in 2012. At present, there are three sublingual tablet immunotherapy products on the market in Canada (Table 1). There will be other allergens for SLIT available soon. The sublingual route of immunotherapy offers multiple potential benefits over the subcutaneous route including the comfort of avoiding injections, convenience of home administration and a favourable safety profile. In addition, SLIT tablets appear to be economically favourable to standard drug therapy, and possibly also to SCIT [6, 7]. This position statement discusses SLIT tablets only, as SLIT drops are not approved by Health Canada.

**Table 1:** Health Canada approved sublingual immunotherapy tablets [8–10]

	Extract composition	Age indication (years)	Dose initiation	Timing of initiation before pollen season	Daily dose
Oralair®	5 grass pollen	5–50	3 day escalation	8–16 weeks	300IR
Grastek®	Timothy grass pollen	≥ 5	Full dose	At least 8 weeks	2800 BAU
Ragwitek®	Short ragweed pollen	18–65	Full dose	At least 12 weeks	12 Amb a 1-U

## How effective is SLIT?

To date there have been many studies evaluating the efficacy of SLIT in management of allergic rhinoconjunctivitis. A Cochrane review, initially published in 2003 and updated in 2011, looked at 60 randomized control trials, including a meta-analysis of 49 studies. Significant reductions in both symptom scores and medication requirements were seen with SLIT compared to placebo [11]. By 2013 a more extensive meta-analysis was published by Lin et al. in the Journal of the American Medical Association including 63 studies and 5131 participants [12]. The vast majority of studies included a single allergen–most commonly grass or dust mite. A benefit of SLIT over placebo was seen in 94% of the studies. Despite the heterogeneity of studies, the strength of evidence was deemed “moderate” in support of SLIT use in allergic rhinoconjunctivitis. Twelve of 13 studies looking specifically at conjunctivitis symptoms showed an improvement with SLIT over placebo. This echoes the findings of a Cochrane meta-analysis focused on the use of SLIT in allergic conjunctivitis published in 2011 and including 42 trials [13]. While no reduction in eye drop use was seen, this study revealed a significant reduction in ocular symptom scores and an increase in threshold dose for conjunctival immediate allergen sensitivity.

Similar strength of evidence was seen to support use of SLIT in children in the 2013 systematic review by Lin [12]. This evidence was based on 9 studies with 471 participants, and was deemed moderately strong to support SLIT use for treatment of rhinoconjunctivitis in this population.

A recent meta-analysis looking specifically at the benefits of SLIT in allergic asthma included 16 trials and 794 patients [14]. A significant reduction in both symptoms and medication scores with SLIT compared to placebo was seen. In the 2013 meta-analysis by Lin, 13 studies looked at asthma control in dust mite SLIT. A statistically significant improvement in asthma symptoms was seen, and deemed to be of strong magnitude by the reviewers [12].

SLIT has been shown to have a sustained benefit once treatment has been discontinued, supporting its disease modifying properties. One 2013 study demonstrated sustained efficacy in the year post-treatment after 3 years of pre- and co-seasonal treatment with a 5-grass pollen sublingual tablet [15]. Durham and colleagues also demonstrated sustained efficacy 2 years after completion of 3 years of pre-seasonal Grastek^®^ treatment [16]. Most studies of SLIT have looked at treatment for a single allergen. Very little data is available regarding multiallergen SLIT in polysensitized individuals [17]. While there are few studies directly comparing the efficacy of SLIT and SCIT, a 2013 meta-analysis indirectly compared systematic reviews. As expected from prior studies, both had significant benefits over placebo, however one modality could not conclusively be deemed superior to the other [18].

## How safe is SLIT?

At the time of the Cochrane review update in 2011, 49 studies had shown a common occurrence of local side effects with SLIT, with no reports of severe systemic reactions, anaphylaxis or epinephrine use. While only 15 studies reported drop-out due to adverse reactions, this was seen in 5% of the SLIT group compared to 1% of the placebo group [11]. In the more extensive 2013 systematic review the authors comment on the lack of a standardized grading system for adverse events among studies, and the inconsistent reporting of adverse events. They deem the evidence insufficient to comment on safety, but do note that while local reactions were common, severe systemic reactions were rare, with no reported cases of anaphylaxis [12].

Clinical trials of Grastek^®^ estimated the rate of severe adverse events at 2.9% versus 1% of the placebo population. The most common local reactions were oral pruritus (26.7%), throat irritation (22.6%) and ear pruritus (12.5%) [6]. In two randomized, double-blind, placebo controlled studies of grass tablet immunotherapy published in 2011 including 439 and 345 patients, each reported one use of epinephrine for treatment-related adverse reactions. The former study reported one non-treatment related use in the placebo group, while the latter reported one non-treatment related use in both the placebo and treatment arms [19, 20]. To date there have been no reported deaths attributed to sublingual immunotherapy. Insufficient evidence is available to make recommendations regarding the safety of SLIT in pregnancy, severe autoimmune disease and immune deficiency.

## When should SLIT be prescribed?

Sublingual immunotherapy for a specific allergen is indicated for those whose rhinitis or rhinoconjunctivitis symptoms are triggered by exposure to that allergen, and who have not responded to, tolerated, or are averse to use of conventional pharmacotherapy. Failure of treatment with traditional pharmacotherapy, however, is not an absolute requirement for use of SLIT. Patients require evidence of sensitization to the relevant allergen via skin prick or in vitro testing. While SLIT has been shown to be safe and effective in children as young as 5, currently only the grass pollen extract products have been approved for use in children [8–10, 12].

Sublingual immunotherapy is contraindicated in patients with severe, unstable or uncontrolled asthma. We advise against use in patients on beta-blocker therapy and in those with active oral inflammation or sores [8–10].

We recommend SLIT only be administered using Health Canada approved products (Table 1).

## Who should prescribe SLIT?

Management of allergic disease requires a collaborative approach between primary care physicians and allergy subspecialists. Primary care physicians should be educated in the detection of allergic disease and be able to identify those patients that could benefit from subspecialty assessment to assist with diagnosis and treatment. Prescribing of SLIT should be limited to recognized allergy subspecialists. Allergy subspecialists should work with the primary care physicians within their referral networks to determine an optimal strategy for re-starts of pre-seasonal SLIT in subsequent years after treatment has already been initiated. Further research would be required before including the prescribing of SLIT as a component of routine primary care practice.

Treatment should be initiated 8–16 weeks prior to, and continue through to the end of, the pollen season (Table 1). For all available SLIT tablet products, the patient should take the first dose under observation in the prescribing physician’s office and monitored for 30 min. The first dose for each season should be given under physician supervision as well. Subsequent doses are self-administered at home, with no food or drink for 5 min after each dose. The current available SLIT tablet products in Canada are initiated at full dose, or with a short 3-day escalation, depending on the product [8–10].

Successful therapy relies on patient adherence to the home regimen. Currently all available product monographs advise returning to the prescribing physician for re-initiation if more than 7 days of therapy are missed. Clear instructions should be given to the patient not to take extra doses if a dose is missed [8–10]. While some physicians may choose to equip those at increased risk for reaction with an epinephrine auto-injector, this is not an absolute requirement for SLIT administration, and should be left to the discretion of the individual allergist and the patient.

In summary, SLIT is an effective modality of treatment for allergic rhinitis and rhinoconjunctivitis. Likely similar to SCIT in efficacy, it can provide long-term benefit with a potentially more favourable side effect profile and increased patient acceptance.

## Key messages

1. Sublingual immunotherapy (SLIT) has been shown to be effective in the management of allergic rhinitis and conjunctivitis.
2. Sublingual immunotherapy is indicated for patients with allergic rhinitis and/or conjunctivitis with evidence of sensitization to the relevant pollen via skin prick test or in vitro testing. It is particularly useful for those who have not responded to or tolerated conventional pharmacotherapy; however, failure of treatment with pharmacotherapy is not an absolute requirement for use of SLIT.
3. SLIT has been shown to be safe in children as young as 5 years of age. While local side effects are common, severe systemic reactions are rare, with no attributed fatalities to date.
4. SLIT has advantages over standard pharmacotherapy in that it may offer disease modification and the potential for long term remission.
5. SLIT offers multiple benefits over the subcutaneous route, including the comfort of avoiding injections, convenience of home administration, and a favourable safety profile.
6. SLIT should only be prescribed by recognized allergy subspecialists.
7. Only Health Canada approved tablets should be used for SLIT.
